# Out-of-pocket expenditure, need, utilisation, and private health insurance in the Australian healthcare system

**DOI:** 10.1007/s10754-023-09362-z

**Published:** 2023-10-11

**Authors:** Timothy Ludlow, Jonas Fooken, Christiern Rose, Kam Ki Tang

**Affiliations:** 1 School of Economics, The University of Queensland, St Lucia, Australia; 2 Centre for the Business and Economics of Health, The University of Queensland, St Lucia, Australia; 3 Macquarie Centre for the Health Economy, Macquarie University, North Ryde, Australia

**Keywords:** Out-of-pocket expenditure, Private health insurance, Chronic conditions, Healthcare need, I13, I14

## Abstract

Despite widespread public service provision, public funding, and private health insurance (PHI), 20% of all healthcare expenditure across the OECD is covered by out-of-pocket expenditure (OOPE). This creates an equity concern for the increasing number of individuals with chronic conditions and greater need, particularly if higher need coincides with lower income. Theoretically, individuals may mitigate OOPE risk by purchasing PHI, replacing variable OOPE with fixed expenditure on premiums. Furthermore, if PHI premiums are not risk-rated, PHI may redistribute some of the financial burden from less healthy PHI holders that have greater need to healthier PHI holders that have less need. We investigate if the burden of OOPE for individuals with greater need increases less strongly for individuals with PHI in the Australian healthcare system. The Australian healthcare system provides public health insurance with full, partial, or limited coverage, depending on the healthcare service used, and no risk rating of PHI premiums. Using data from the Household, Income and Labour Dynamics in Australia survey we find that individuals with PHI spend a greater share of their disposable income on OOPE and that the difference in OOPE share between PHI and non-PHI holders increases with greater need and utilisation, contrary to the prediction that PHI may mitigate OOPE. We also show that OOPE is a greater concern for poorer individuals for whom the difference in OOPE by PHI is the greatest.

## Introduction

Higher life expectancy and an ageing population contribute to an increasing prevalence of chronic conditions in developed countries (Christensen et al., 2009). Chronic conditions increase the need for long-term and acute care and lead to greater expenditure on healthcare (de Meijer et al., 2013; Spillman & Lubitz, 2000). Public provision of services and social insurance, or private health insurance (PHI) and out-of-pocket expenditure (OOPE), can finance this expenditure. OOPE creates a policy concern if it requires a high share of income, particularly among poorer individuals who may face trade-offs between healthcare and other essential goods and services. Policy makers may also consider whether, in their healthcare system, individuals with greater need can protect themselves from large OOPE by purchasing PHI. Here we study individual-level OOPE by PHI, chronic conditions, and hospitalisation to shed light on this potential role of PHI in the Australian healthcare system.

Around 20% of all healthcare expenditure in the OECD is financed through OOPE (Organisation for Economic Co-operation and Development, 2017), and private payments are common even when public health insurance is widespread, or healthcare is publicly provided. Chronic conditions often create a significant financial burden for individuals through greater OOPE, particularly because their prevalence is typically higher among poorer individuals (Lehnert et al., 2011; Carpenter et al., 2015). Additionally, the OOPE burden, measured by the share of available income spent on OOPE, is often regressive in nature, causing an equity concern for population groups that have lower income and chronic conditions (Korda et al., 2014). Thus, OOPE can conflict with equity goals unless adequate protections for higher need and lower income groups are in place (Lairson et al., 1995).

To manage the burden of high OOPE in case of need for care, individuals with a greater risk of OOPE may consider purchasing PHI to smooth their consumption by paying a certain (known) premium instead of uncertain (volatile) OOPE. If PHI providers are limited in their legal or actual ability to charge risk-rated premiums and deny individuals with higher need to enrol in an insurance plan, as it is the case in Australia, higher-need individuals that require more ongoing care or hospital services may also purchase PHI to shift some of their financial burden onto lower-need individuals who purchase PHI for reasons other than healthcare need, such as tax incentives. Hence, Australian PHI may mitigate the OOPE burden for individuals with higher need when using private healthcare services without being required to make additional PHI premiums. However, PHI may also lead to greater use of PHI-financed private healthcare services that are not provided in the public system and require OOPE payments even if individuals have PHI, and to the substitution of public (free) healthcare for private (costly) healthcare. In both of these cases, OOPE may increase.

In this paper, we use Australian data to investigate if the burden of OOPE differs between individuals with and without PHI and whether chronic conditions or hospitalisation increase this difference. This allows us to observe if the increase of OOPE for individuals with chronic conditions and hospitalisation depends on PHI. Additionally, as a large OOPE burden may be more problematic for poorer individuals, we investigate if our results vary by income.

Our paper contributes to the literature by investigating whether PHI in the Australian healthcare system mitigates the increase in the OOPE burden for individuals who require more care due to chronic conditions or hospitalisation. Existing research has shown that chronic conditions are associated with greater healthcare need and increase the burden of OOPE in Australia. McRae et al. (2013) study the impact of multiple chronic conditions on OOPE and find that the share of income needed to cover OOPE increases with the number of chronic conditions for individuals over 50. Several specific chronic conditions and the type of chronic condition have also been shown to be predictors of OOPE (Callander et al., 2017; Essue et al., 2011, 2013). However, while establishing higher need, as indicated by chronic conditions, as a predictor of greater OOPE in the Australian healthcare system, previous research has not considered whether PHI may weaken the link between more utilisation due to greater need and the OOPE burden. Only two prior studies report OOPE by PHI status, considering cancer (Gordon et al., 2018) and rheumatoid arthritis (Lapsley et al., 2002) patients. Both find that OOPE is higher for individuals with PHI. However, no prior study has used population-wide data to consider if the increase in the OOPE burden associated with greater need and utilisation may differ between individuals with and without PHI. Furthermore, prior research has not investigated how higher OOPE due to greater need may vary for different income groups.

Our results show that individuals with PHI spend a greater share of their income on OOPE than those covered by public insurance alone, irrespective of their status of reporting no chronic condition, a chronic condition, or both a chronic condition and hospitalisation. Because PHI premiums are excluded from our measure of OOPE, this greater OOPE burden for PHI holders is in addition to their PHI premium expenditure. Although chronic conditions and hospitalisation are associated with a greater share of income spent on OOPE for both PHI and non-PHI groups, the absolute increase in the OOPE burden is greater for individuals with PHI. This suggests that PHI offers only limited protection against OOPE for individuals who require hospitalisation or use more healthcare services due to their chronic conditions, while potentially offering other benefits such as greater choice or improved access to care.

## Background

The cornerstone of the Australian healthcare system is ‘Medicare’, a universal, tax-funded public health insurance providing full coverage for treatment in public hospitals and full or partial coverage for general and specialist outpatient services. Outside of public hospitals, many services in primary and in specialist care attract a so-called ‘rebate’ (a subsidy as defined in a list price) that is funded by Medicare, but providers are free to set their fees at any level above the rebate. The difference between the provider’s fee and the Medicare rebate is known as the ‘gap’. Patients, therefore, incur OOPE when they seek outpatient care, except when their provider charges exactly the rebate, which is referred to as bulk billing. Importantly, PHI is regulated such that it must not cover gap payments for outpatient care that qualifies for Medicare rebates.

Medicare and other public healthcare entities, such as community healthcare clinics, either provide very limited coverage of ancillary health services (such as dental, optical and physiotherapy) or do not cover them at all. Individuals without PHI covering these ancillary health services must pay the full cost for using them. Furthermore, individuals with PHI may still incur OOPE for these services because their insurance plan is based on list prices and even if their insurance covers 100 percent of the list price (which is not always the case), service providers can charge higher prices, requiring patients to pay the difference between the amount covered by their PHI and the actual price.

Finally, OOPE occurs when individuals choose to seek private hospital care. While care in public hospitals does not incur any OOPE, private hospitals charge fees above the amount reimbursed by Medicare. Costs for private hospital care are covered through PHI, OOPE, or a mixture of PHI and OOPE. Because many PHI plans for hospital care use list prices that are lower than the hospital fee, and therefore only cover part of the fees, and because there can be excess charges, OOPE is typically required for private hospital care, even when PHI and Medicare cover most of the cost.

The Australian healthcare system assigns a central role to PHI. Forty-four percent of adults have coverage for private hospital treatment and fifty-three percent for ancillary services. Although hospital and ancillary cover can be bought separately, they are often purchased together (Australian Prudential Regulation Authority, 2020). PHI reduces the cost of private hospital and ancillary care, but usage of privately provided services typically includes co-payments (Duckett & Nemet, 2019a).^1^ PHI is legally prohibited from covering gap payments and, therefore, cannot reduce OOPE for outpatient services covered by Medicare.

PHI in Australia is legally required to offer open enrolment (i.e., no denial of coverage) and community rating (i.e., not risk rating). This guarantees that the same premium is paid for identical products, regardless of the health status or medical history of individuals who purchase PHI. Theoretically, open enrolment and community rating increase the problem of adverse selection, as high-need individuals may self-select into PHI and low-need individuals out of it. However, government policies introduced in the late 1990s and early 2000s in response to declining rates of PHI coverage create strong incentives for young and high-income (i.e., low-risk) individuals to purchase PHI. The policies include the Medicare Levy Surcharge (MLS), a levy on income that individuals who surpass a certain income threshold and do not have at least a minimal level of PHI coverage for hospital care must pay. Because the MLS penalty for individuals with incomes above the MLS threshold is greater than the cost of the lowest-cost PHI plan that allows to avoid the penalty, individuals have a strong incentive to purchase PHI for hospital care. The Lifetime Health Cover (LHC) is a second policy to strengthen the effect of the MLS. LHC specifies that individuals who do not have PHI for hospital care and begin purchasing hospital PHI after their 31^st^ birthday must pay higher PHI premiums for every year that they delay taking up PHI after turning 31. Individuals who do not surpass the MLS threshold at age 31 but expect to do so later in their life are therefore incentivised to purchase hospital PHI when they turn 31. In addition, there are subsidies (called ‘rebates’) for both hospital and ancillary PHI. Because hospital and ancillary PHI are often purchased in a bundle, these policies increase the uptake of both hospital and ancillary PHI. However, the MLS and the LHC only apply to PHI that covers private hospital care. Hence, the policies, particularly the MLS and LHC, provide strong incentives for economically advantaged individuals, who also tend to be healthier, to purchase PHI. As a result, individuals with PHI have better health than those without PHI, with some research suggesting that the Australian PHI market is better described by advantageous rather than adverse selection (Buchmueller et al., 2013).

Hospital and ancillary care use leads to significant OOPE in Australia. In 2019–20, individual health spending on dental and other outpatient healthcare services was about $7 billion and individual health spending on hospitals was about $3 billion (Australian Institute of Health and Welfare, 2021). While considering that both hospital and ancillary services require OOPE, it is important to recognise that the potential impact of PHI on individual-level OOPE differs between the two. Generally, ancillary services are poorly covered in the public system, exposing non-PHI individuals to their full cost. This is particularly important for individuals with chronic conditions, many of whom having an ongoing need for ancillary services, such as physiotherapy. PHI cover should, therefore, protect against OOPE from ancillary services, even in the presence of co-payments, because individuals shift their spending for these services from variable OOPE to fixed PHI premiums.

By contrast, private hospital services largely duplicate (non-emergency) parts of the free public hospital system, while offering additional benefits such as reduced waiting times, choice of physician, and better amenities. Individuals with PHI who are hospitalised as private patients may have increased exposure to OOPE, particularly if their policy includes significant co-payments or if there are excess charges, which is very often the case (Australian Institute of Health and Welfare, 2017). By comparison, individuals without PHI treated as public patients do not face any co-payments. Importantly, however, private hospital insurance does not require individuals to opt out of the public system. Hence, individuals who have PHI for hospital care can utilise public hospital treatment if they choose to. Furthermore, individuals without PHI may self-insure and use private hospital care. However, the latter case is uncommon because the high cost of private care and the ability to use free public care create a large cost differential between these two options for individuals without PHI.

Hence, PHI may reduce OOPE for ancillary services, even if some degree of moral hazard leads to greater use of those services. The case is more complicated for PHI covering private hospital care. Hospital PHI may reduce OOPE for those using the private hospital but could also increase OOPE because it is attained by individuals who plan to opt into treatment in private hospitals. Use of private hospital care will lead to higher OOPE relative to using public hospital care, and individuals with hospital PHI may be more likely to use private hospitals because their PHI lowers the associated OOPE, making private hospital care relatively more affordable. Hence, while PHI for hospital care reduces the cost of treatment in private hospitals, it may be associated with higher OOPE because it allows individuals to use care that requires OOPE, as opposed to using public hospitals, which do not have user charges.

## Method

### Data

We use data from the Household Income and Labour Dynamics in Australia (HILDA) survey, which is representative of the Australian population and has followed more than 17,000 individuals each year since 2001. HILDA collects detailed information on income, labour, and family dynamics. It has been used to study various aspects of health, including OOPE (Callander et al., 2019), private health insurance (Buchmueller et al., 2021), and inequity in health and healthcare utilisation (Kessels et al., 2020; Fooken & Jeet, 2022). The survey includes a health module containing a range of health-related questions that is administered in every fourth wave since in 2009. We use this health module data from years 2009, 2013, and 2017.^2^

We further restrict our analysis to a subsample of this data that comprises of a population with greater healthcare need. First, we limit our sample to adults over 40 years of age, as our variable indicating greater healthcare need is the presence of chronic conditions, which tend to increase with age and are more prevalent later in life. Furthermore, our utilisation variable, hospitalisation, may be affected by strong preferences for using private hospital services for maternity care, which may constitute a special case concerning PHI-funded hospital care. As most women giving birth are under the age of 40, we chose this age as our cut-off. Second, we restrict our sample to individuals living in households that have less than five people. This is because healthcare expenditure is collected at the household level, and expenditure and resource pooling in larger households may influence the results.

#### Variables

HILDA data include three healthcare expenditure measures: Fees paid to health practitioners; expenses for medicines, prescriptions, and pharmaceuticals; and PHI premiums. Healthcare expenditure data are collected at the household level. We, therefore, use the household-level expenditure as an individual-level variable. Robustness checks that conduct the analysis by different types of households (see appendix) indicate that this modelling decision has no significant influence on our results. We chose individuals over households as the unit of analysis because household composition is not always stable over time.

To construct our primary outcome variable, the share of disposable income used to pay for OOPE, we first calculate the sum of fees paid to health practitioners and expenses for medicines, prescriptions, and pharmaceuticals. We subsequently derive the OOPE share by dividing OOPE by disposable household income and express this share as a percentage, as is common in research on the burden of OOPE (Baird, 2016; Callander et al., 2019; Al-Hanawi, 2021). That is, equivalent levels of OOPE will create a greater burden for individuals with less income. Furthermore, we adjust the disposable income variable by household size, using OECD Modified Equivalence Weights (Organisation for Economic Co-operation and Development, 2020) when household disposable income is used as a regressor. The resulting variable, which we refer to as *disposable income* in the following, is also inflation-adjusted and expressed in constant 2017 dollars.

We define *Chronic Condition* as a composite variable that is based on two measures included in HILDA. The first measures if the individual has been told by a doctor or nurse that they have any chronic condition (from a list presented to the survey respondent) that has affected them in the past year. The second describes if the individual has any long-term health conditions, impairments, or disabilities, that limit their activities and has lasted, or is expected to last, for longer than six months. *Chronic Condition* is indicated by a positive response to either of these two survey questions.

We define *Hospitalisation* as any day or overnight hospitalisation in the past year.

Based on the *Hospitalisation* and *Chronic Condition* variables, we subsequently define a *Lower Need* group that includes individuals with no chronic condition and no hospitalisation, a *Higher Need* group that includes individuals reporting a chronic condition and no hospitalisation, and *Higher Need with Hospitalisation* that includes individuals with both a chronic condition and at least one hospitalisation. We drop respondents with no chronic conditions but at least one hospitalisation from our sample.

We note that our definition of *Need* is based on a diagnosis (chronic condition), which differs from commonly studied need as described by demographic, socioeconomic, and health status variables. Furthermore, hospitalisation only describes utilisation and therefore does not necessarily reflect greater need. However, hospitalisation is correlated with increased need because individuals require referrals for both private and public hospital care and, besides needing a referral, there are no substantial barriers to accessing hospitals in the public system. We chose these three categories because they allow us to define a gradient of need and utilisation that may affect OOPE from the use of ancillary and hospital services, the two types of care that are covered by PHI in Australia.

Our definition of PHI is based on a survey item that elicits whether individuals have no PHI, PHI for hospital care only, PHI for ancillary care only, or PHI for both hospital and ancillary care. We focus on those either with no PHI or with PHI for both hospital and ancillary care, who constitute the two largest groups in the survey. We do not consider individuals with only ancillary or only hospital PHI because we do not know how comprehensive their coverage is and cannot define which individuals in these two groups have more or less access to PHI-funded private care. Therefore, for our sample, we define an individual to have PHI if they are covered by both hospital and ancillary care and not to have PHI when they are covered for neither.^3^

We also include a set of control variables in our estimations. These include the respondent’s age, disposable income, the highest level of education (year 12, certificate or diploma, bachelor degree, postgraduate degree, and the baseline of year 12 or less), an indicator of whether the respondent has a health care card, the type of household the respondent lives in (couple without children, couple with children, single parent, other household, and the baseline of lone person households), the number of children and the presence of children under 5 years of age in the respondent’s household, the respondent’s location of residence (inner regional, outer regional, remote, and major city as the baseline), and the respondent’s health using the physical health component score (PCS) from the Short Form 36 survey. The PCS score uses a weighted combination of answers to the 36 questions of the Short Form 36 survey and provides a (self-reported) measure of the physical health of respondents.

Finally, we adjust our data by removing potential outliers. A small number of observations report multiple standard deviations above the mean OOPE, and some observations for disposable income are negative. We remove observations if they are in the bottom 1% of disposable income, including very low reported disposable incomes, sometimes reporting values of less than $500 per year, an income that does not appear sufficient for subsistence level living in Australia. We also remove the top 1% of OOPE share. After these restrictions, our main sample consists of 19,786 observations from 9,754 individuals. A few estimations include slightly smaller numbers of observations (and individuals) when there are missing values for some of the control variables included in the estimation.

#### Summary statistics

Table 1 shows the sample summary statistics by our three categories of lower need, higher need, and higher need with hospitalisation, with the data pooled across the years 2009, 2013 and 2017. Twenty-nine percent of our sample have lower need, 48% higher need, and 23% higher need and hospitalisation. The average age is 54 years for individuals with lower need, 61 years for individuals with higher need, and 65 years for individuals with higher need and hospitalisation. The average OOPE share is 1.6% of disposable income for individuals with lower need, 2.3% for individuals with higher need, and 3.1% for individuals with higher need and hospitalisation. Hence, the OOPE share increases with need and hospitalisation. We note that the average level of OOPE share does not appear to be excessively high, even for the groups with the greatest OOPE share. Disposable income is lower for individuals with greater need and hospitalisation, falling from $63,624 for individuals with lower need to $49,084 for individuals with higher need and hospitalisation.

**Table 1:** Summary statistics by need and hospitalisation

	Lower need			Higher need			Higher need and hospitalisation		
	Obs	Mean	SD	Obs	Mean	SD	Obs	Mean	SD
OOPE share (%)	5753	1.58	2.35	9446	2.3	2.95	4587	3.12	3.8
PCS score_t-1_	5594	50.13	10.43	9223	42.45	12.45	4468	37.47	12.67
Age (in years)	5753	53.51	9.65	9446	61.35	12.18	4587	64.74	12.57
Disposable Inc ($)	5753	63624	42324	9446	50921	47501	4587	49084	57294
PHI	3432	60%		4789	51%		2410	53%	
Health care card	998	17%		4832	51%		2875	63%	
Less than Yr 12	1337	23%		3606	38%		1886	41%	
Yr 12	573	10%		812	9%		352	8%	
Certificate	2031	35%		3060	32%		1489	33%	
Bachelor	917	16%		955	10%		432	9%	
Postgraduate	893	16%		1005	11%		422	9%	
Lone Person	940	16%		2110	22%		1202	26%	
Couple; No Child	2903	50%		5338	57%		2652	58%	
Couple; Children	1445	25%		1097	12%		335	7%	
Single Parent	365	6%		635	7%		279	6%	
Other	99	2%		265	3%		119	3%	
No child	4284	74%		8351	88%		4228	92%	
1 child	786	14%		683	7%		227	5%	
2 children	677	12%		408	4%		131	3%	
3 children	6	0%		4	0%		1	0%	
Major city	3570	62%		5477	58%		2590	56%	
Inner regional	1448	25%		2589	27%		1296	28%	
Outer regional	611	11%		1207	13%		595	13%	
Remote	122	2%		172	2%		106	2%	

The sample consists of individuals over the age of 40 years who live in a household with no more than four members who have either no or both ancillary and hospital PHI. ‘Disposable Inc’ is household regular disposable income, equalised for the household size. Children are defined as being under the age of 15 years. Education indicates the highest attained level of education. ‘Obs’ indicates the number of observations and ‘SD’ the standard deviation

**Fig. 1:**
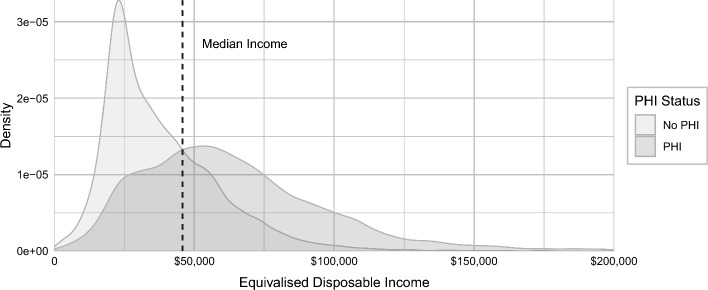
Distribution of disposable income by PHI status. *Notes* Density estimates are of the regular disposable household income in 2017 after being equivalised for household size. The vertical dashed line is the sample median disposable income of $$\$47,060$$; this is the median for all individuals, regardless of PHI status. The x-axis is limited to disposable income values below $200,000. The densities are produced by Gaussian kernel density estimation using the ‘rule-of-thumb’ bandwidth selection

Table 1 also highlights that the fraction of individuals with PHI does not markedly increase with need and hospitalisation, as would be predicted if adverse selection were prevalent in the Australian PHI market. With 60% of individuals with lower need, 51% of individuals with higher need, and 53% of individuals with higher need and hospitalisation reporting to have PHI, there is no clear gradient of the probability of having PHI across our categories. Furthermore, Fig. 1 shows the distribution of disposable income by PHI status and reveals a significant overlap between PHI and non-PHI groups in terms of their income. Although individuals with PHI generally have higher incomes, 34% of individuals with below-median income have PHI. Hence, both higher and lower income groups include a significant proportion of individuals with PHI.

### Statistical analysis

We use two approaches to predict the OOPE share conditional on PHI and across our three categories of lower need, higher need, and higher need with hospitalisation. The first predicts the OOPE share using a random effects model, which accounts for repeated observations at the individual level over time.^4^ The second approach predicts the OOPE share across the distribution of disposable income in the population using the ‘Robinson’s double residual’ estimation method (Robinson, 1988). Because the estimated relationship between OOPE and income provides an estimate of the responsiveness of the share of income spent on OOPE to income changes, it represents a ‘statistical Engel curve’ and is referred to as the Engel curve from here onwards.^5^

Both methods allow us to predict the conditional mean of the OOPE share but have different advantages. The random effects model provides more efficient estimates by taking advantage of repeated observations by individuals. The Engel curve estimation allows us to estimate the relationship between the OOPE share and income without the need to specify the functional form of their relationship. By comparison, an analysis of the OOPE share across the income distribution in the random effects model would require additional assumptions regarding the relationship between the OOPE share and income (e.g., to have a linear, log-linear, or quadratic relationship). For both approaches, predictions of the OOPE share are made at the within-group mean for all control variables, where the groups are defined by our three categories of lower need, higher need, and higher need with hospitalization. The only exception is the variable disposable income, for which we predict the OOPE share at the median because the long right tail of the income variable implies that the mean is less informative about the income of most sample members than the median.

#### Random effects model

To estimate the OOPE share by PHI and our three categories of lower need, higher need and higher need with hospitalisation, we use the following random effects model:

1 $$\begin{aligned} OOPES_{it} = \;&\alpha _i \;+\; \delta _1\; Higher_{it} + \delta _2 \; HigherHosp_{it} + \gamma _0 \; PHI_{it} \;+ \nonumber \\&\gamma _1 \left( PHI_{it} \times Higher_{it} \right) + \gamma _2 \left( PHI_{it} \times HigherHosp_{it} \right) + X_{it}^{\prime } \beta + \epsilon _{it}, \end{aligned}$$

where $OOPES_{it}$ is the OOPE share for individual *i* in period *t*, $Higher_{it}$ is a binary indicator equal to one if individual *i* has higher need in period *t* and zero otherwise, $HigherHosp_{it}$ is a binary indicator for higher need with hospitalisation, and $\alpha _i$ is an individual level random effect that captures heterogeneity between individuals. Note that *LowerNeed* and its interaction with PHI are not included in equation (1), hence, coefficients are interpreted with reference to the baseline of *LowerNeed*.

The vector $X_{it}$ includes the following control variables: The natural logarithm of disposable income, denote as *inc*; an indicator equal to one if *i* holds a health care card in period *t*; indicator variables for the highest attained level of education; indicator variables for the household type that individual *i* belongs to; indicators for the number of children in the household^6^; an indicator equal to one if there are children under 5 years of age in the household; indicators for the area of remoteness; and calendar year fixed effects. We also consider two additional variables in $X_{it}$ that control for health status differences, namely the first lag of the PCS and age. The PCS is lagged by one year so that both the PCS and age are predetermined.

In our results, we report three estimation specifications with different sets of control variables. The first includes no control variables, the second includes all control variables except for PCS and age, and the third includes the full set of control variables.

When estimating equation (1), the random error term $\epsilon _{it}$ is assumed to have a zero conditional mean across all *i* and *t*, such that $\mathbb {E}\left[ \epsilon _{is}|W_{it},\alpha _i\right] =0$ for all *s* and *t* in $\{2009, 2013, 2017\}$, where $W_{it}$ includes all covariates in equation (1). We report cluster robust standard errors, with clustering at the individual level.

#### Engel curves

Because the (in)equitable distribution of OOPE across the population by economic disadvantage largely determines whether OOPE poses a policy concern, we further investigate the burden of OOPE across different income groups. We do so by estimating the mean of the OOPE share across the distribution of disposable income. Income can be seen as an indicator of inequity, as poorer individuals should not be those exposed to a greater burden of OOPE. The relationship between the share of expenditure for a particular good (the OOPE share in our case) and income is often referred to as an ‘Engel curve’. We model our Engel curve allowing for a nonlinear relationship between the OOPE share and disposable income.^7^

The following model of a statistical Engel curve describes our approach for a given PHI status and a given level of our three categories of lower need, higher need and higher need with hospitalisation:

2 $$\begin{aligned} OOPES_{j} = \alpha + g\left( inc_{j} \right) + x_{j}^{\prime } \theta + u_{j}, \end{aligned}$$

where $g(\cdot )$ is an unspecified (possibly nonlinear) function. We use subscript *j*, as opposed to *i*, to highlight that the three time periods are pooled in equation (2).^8^ The vector $x_{j}$ contains the full set of control variables as listed for $X_{it}$ in equation (1) except $inc_j$. The random error $u_{j}$ is assumed to satisfy the zero conditional mean assumption $\mathbb {E}[u_j|inc_j, x_j]=0$. Equation (2) describes a *partially linear model*, and we estimate $g\left( inc_{j} \right)$ and $\theta$ using the *double residual* approach (Robinson, 1988). We estimate six Engel curves by conditioning the sample on each of the three categories of lower need, higher need and higher need with hospitalisation, all of which can be observed with and without PHI, creating six possible combinations. We bootstrap pointwise confidence intervals around $g\left( inc_{j} \right)$ using 1,000 bootstrap samples.

## Results

### Random effects predictions

**Fig. 2:**
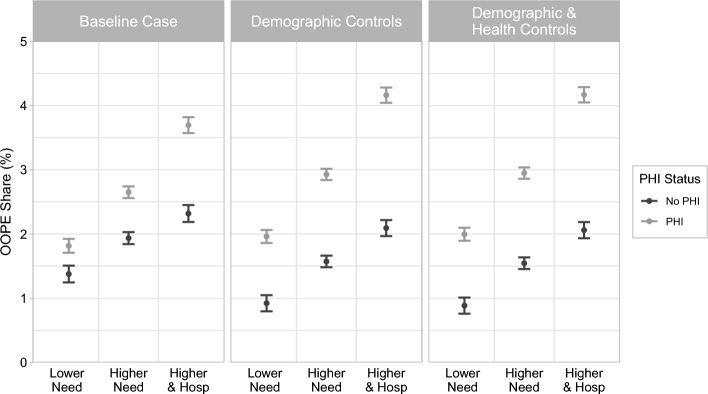
Random effects predictions by need, hospitalisation and PHI status. *Notes* Error bars around the point estimates display predicted 95% confidence intervals. The ‘Baseline Case’ predictions are without control variables. The ‘Demographic Controls’ predictions include the following control variables: the natural logarithm of (equivalised) regular disposable income, highest level of education, household type, number of children in household, children under five years, and remoteness region of residence. The ‘Demographic & Health Controls’ additionally includes age and the previous year’s health status (the first lag of the PCS). Predictions are made at the mean of all control variables, except for the income, where the log of the median disposable income is used

Figure 2 shows the predicted conditional mean of the OOPE share (with 95% confidence intervals), based on equation (1). The left panel describes estimates without control variables, the middle panel estimates with all controls except for the PCS and age, and the right panel includes the full set of controls. Figure 2 visualises our main results. A higher predicted OOPE share is associated with greater need and hospitalisation and is higher for individuals with PHI.

Table 2 provides the numerical estimates corresponding to the right panel of Fig. 2 and quantifies the difference in the OOPE share by PHI status. Appendix Table 4 includes all the estimated coefficients based on equation (1). The predicted OOPE share of individuals with lower need and no PHI is 0.92%, 1.57% for those with higher need and no PHI, and 2.09% for those with higher need with hospitalisation and no PHI. The OOPE share is greater for individuals with PHI than those without in each of our three categories and their predicted OOPE share also increases with need and with hospitalisation. The predicted OOPE share of individuals with PHI and lower need is 1.96%, 2.93% for those with PHI and higher need, and 4.16% for those with PHI and higher need with hospitalisation. The bottom row of Table 2 shows that the difference in OOPE share between individuals with and without PHI increases with greater need and hospitalisation. Hence, individuals with PHI observe a higher OOPE share across all of our three categories of need and hospitalisation. The increase in OOPE associated with greater need is greater for individuals with PHI.

**Table 2:** Random effects predictions of the OOPE share (%) by PHI status, need, and hospitalisation

Model: Conditional mean (random effects)			
PHI status	Lower need	Higher need	Higher need and hospitalisation
No PHI	0.92	1.57	2.09
	(0.8, 1.05)	(1.48, 1.66)	(1.97, 2.22)
PHI	1.96	2.93	4.16
	(1.86, 2.06)	(2.84, 3.02)	(4.04, 4.28)
Difference	1.04	1.36	2.07

Predictions of the conditional mean estimates using the random effects model with the full set of control variables. The 95% confidence intervals are presented in brackets, below the point estimates. The ‘Difference’ row presents the OOPE share difference (*pp*) between individuals with and without PHI, within each category defined by need and hospitalisation

Comparing our results between the three panels of Fig. 2 also illustrates that our control variables do not drive these relationships. In the appendix we include further robustness analysis showing that our results are also replicated when splitting our sample by those under and over 65 years of age and when estimating the OOPE share separately for different types of households (single individuals, couples with no children, couples with children, single parents, or other).

Our results also provide estimates for the average level of OOPE burden. Individuals without PHI spend, on average, a share of less than 1% of their income on OOPE when they have lower need. Although the OOPE share increases with need, we note that even individuals with higher need and hospitalisation report an average share of income used to pay for OOPE that is not much greater than 2%. The values are higher for those with PHI, but even for individuals with higher need and hospitalisation, an OOPE spending of 4.2% of income on average does not appear overly high. Hence, while the relative differences and the relative increase in the share of income spent on OOPE by our categories of analysis are large, policy makers may not be too concerned about the average levels of the burden of OOPE, as these appear moderate, provided individuals are not severely income constrained. We next turn to our Engel curve analysis to observe how informative these averages are across different income groups.

### Engel curve predictions

**Fig. 3:**
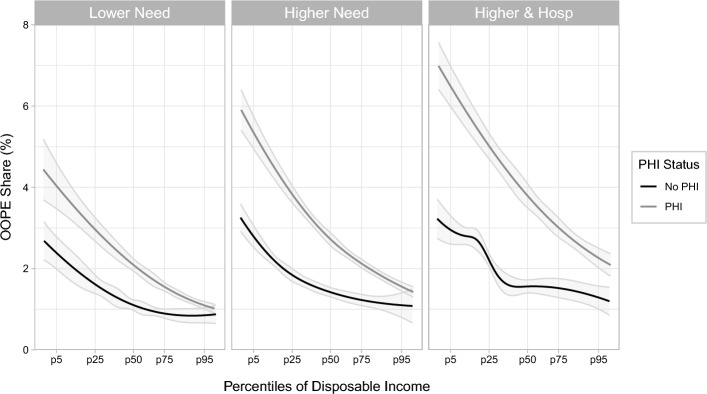
Engel curves by PHI, need, and hospitalisation. *Notes* Engel curves are estimated separately after first partitioning the sample by PHI, *Need, and Hospitalisation*. Estimates are performed using the full set of control variables. The grey shaded regions around the Engel curves are $95\%$ confidence intervals, estimated using 1, 000 bootstrapped samples

Figure 3 shows the estimated Engel curves by PHI status for lower need (left panel), higher need (middle panel) and higher need with hospitalisation (right panel). The x-axis includes indicators for the percentiles of disposable income (the 5th, 25th, 50th, 75th and the 95th). All our Engel curves are downward sloping, indicating that the burden of OOPE, as measured by the share of OOPE relative to income, is greater for individuals with lower incomes. This result is observable both for individuals with and without PHI.

Table 3 adds numeric predictions of OOPE share for the 25th, 50th, and 75th income percentiles. At each percentile, the Engel curve results are qualitatively the same as the random effects predictions. The OOPE share is greater for individuals with PHI than for those without PHI at each level of need and utilisation, and this observation is true across the entire distribution of disposable income. Furthermore, corresponding to our results based on the random effects model, the differences in the OOPE share between those with and without PHI increase with need and utilisation (from the left to the right panel of Fig. 3). Our Engel curves also illustrate that the absolute difference increases most strongly at the lower income percentiles. Hence, increasing need and hospitalisation are particularly associated with higher OOPE shares in the median and lower income groups with PHI.

**Table 3:** Engel curve predicitons of the OOPE share (%)

Model: Conditional mean (engel curve)				
Income percentile	PHI status	Lower need	Higher need	Higher need and hospitalisation
25th	No PHI	1.62	1.84	2.15
		(1.39, 1.85)	(1.69, 2)	(1.99, 2.31)
	With PHI	2.95	3.8	4.98
		(2.66, 3.23)	(3.6, 4)	(4.7, 5.29)
	Difference	1.33	1.96	2.83
50th	No PHI	1.1	1.42	1.56
		(0.97, 1.23)	(1.31, 1.53)	(1.4, 1.72)
	With PHI	2.11	2.7	3.79
		(1.96, 2.27)	(2.55, 2.86)	(3.49, 4.09)
	Difference	1.02	1.28	2.23
75th	No PHI	0.89	1.23	1.52
		(0.78, 1.01)	(1.09, 1.37)	(1.29, 1.75)
	With PHI	1.56	2.13	3
		(1.45, 1.66)	(2.02, 2.23)	(2.76, 3.24)
	Difference	0.66	0.9	1.48

Engel curve predictions are from the 25th, 50th, and 75th percentiles of the sample equivalised disposable income. Bootstrapped 95% confidence intervals are presented in brackets below the point estimates

## Discussion

### PHI mitigating OOPE for higher-need individuals

Our results show that the OOPE share increases for individuals with chronic conditions and when they have chronic conditions together with hospitalisation. These findings are in line with prior research finding that (particularly multiple) chronic conditions are strong predictors of high OOPE (McRae et al., 2013; Lehnert et al., 2011), and that chronic conditions and OOPE are positively related (Essue et al., 2011, 2013). Policy makers and individuals may consider whether supplementing public health insurance and healthcare provision with PHI may be a suitable option to mitigate the increase in OOPE when need increases and when hospitalisation is required. PHI may be a particularly appealing option for such an approach in Australia because regulation ensures that individuals with greater need cannot be excluded from PHI and do not face higher premiums, reducing equity concerns regarding potential access to PHI.

However, contrary to this possible role of PHI, the existing literature suggests that OOPE tends to be higher for individuals with PHI (Lapsley et al., 2002; Gordon et al., 2018) and our results align with these prior results. We find that the share of income used to pay for OOPE is consistently higher for individuals with PHI and that the difference in this OOPE burden between individuals with and without PHI increases with need and with hospitalisation, in contrast to the suggestion that PHI may mitigate the burden of OOPE. Hence, PHI enrolment appears to be a predictor of greater OOPE, despite the fact that PHI covers some of the cost of seeking private health care. This is because individuals with PHI use more healthcare services that require OOPE than individuals without PHI.

### Adverse selection and sorting

Before concluding that PHI does not mitigate the risk of exposure to OOPE, it must be considered that differences in OOPE by PHI may be the consequence of adverse selection. That is, individuals with private information about their greater need for healthcare services may be more likely to purchase PHI. However, previous research suggests that the Australian market for PHI is described by advantageous, not adverse selection (Buchmueller et al., 2013). Similarly, our results do not appear to be driven by adverse selection because the difference in the OOPE share by PHI is unaffected by controlling for potential sources of adverse selection. That is, when we include in our estimations additional control variables that are predictors of need known to the insurance buyer but cannot be used by the insurer to risk-rate premiums (e.g., location of residence, PCS, and age), our results do not change substantially. This indicates that there is no significant adverse selection based on these observable variables.

One may also argue that community rating and open enrolment may encourage individuals with ‘bad risks’ – individuals with private information about their greater need – to purchase PHI and these risks may be unobserved. These higher-need individuals with PHI may then still face greater OOPE than non-PHI individuals because PHI only partially covers additional treatment costs. Hence, PHI reduces the difference in OOPE between higher need individuals with PHI and lower need individuals without PHI. However, the pool of individuals with PHI still incurs greater OOPE because it comprises of individuals with significantly greater need. Yet, while this explanation may rationalise the overall differences in OOPE by PHI, it does not explain our finding that the difference in the OOPE share by PHI status increases with the level of observed need and with hospitalisation. That is, there should be no greater selection in and out of PHI for individuals with greater observed need or utilisation because, in the presence of community rating and open enrolment, these two factors do not influence the premium an individual must pay for PHI. Greater selection would only be expected if observed need and utilisation are correlated with unobserved need, which does not appear to be the most likely case, given that individuals with greater observed need and utilisation are likely to use more healthcare services, which makes it less likely that a given need remains unobserved (e.g., undiagnosed).

Individuals may still sort in and out of PHI due to their expected need, but this sorting should not increase with need and utilisation because community rating prevents PHI premiums from increasing with need and utilisation. However, while community rating prohibits levying higher costs from greater utilisation on higher-need insurance takers via the premium, it could be levied on individuals with greater need and utilisation through higher co-payments. Higher co-payments could be levied by limiting the number of healthcare services (e.g., the number of physiotherapy sessions) covered or the coverage of a policy (e.g., the percentage of costs covered by the insurance). Restrictions are very common in Australian PHI policies. Reducing all potential restrictions for all unknown future health need may be increasingly expensive relative to buying a basic level of coverage. This could lead to an implied risk-rating through OOPE instead of through premiums and could explain why PHI does not appear to mitigate the burden of OOPE for individuals with higher need and higher utilisation.

### Moral hazard

Our results indicate that the OOPE share increases with need and hospitalisation more strongly for those with PHI than those without. One explanation for this finding may be that PHI leads to moral hazard because it reduces the cost of care. PHI holders may consume more healthcare services or use private hospital care instead of public hospital care because these are less costly with PHI. How PHI reduces the cost of care is apparent for ancillary services, for which there is no substitute in the public system.^9^ The case is different for PHI for hospital care because PHI reduces the cost of private hospital care, reducing the additional OOPE required when using the private hospital instead of the public hospital. Still, the use of private hospitals will continue to require greater OOPE than using public hospitals.

Yet, if PHI is an insurance in the sense of allocating uncertain variable expenditure (OOPE) to certain fixed expenditure (PHI premiums) for care, individuals should not be exposed to greater OOPE, even with greater consumption of (now cheaper) healthcare services due to PHI. This is particularly the case for PHI for ancillary care, where public healthcare is not necessarily available, and for very comprehensive private hospital care that does not require any or only minimal co-payments at the point of consumption. That is, moral hazard alone may lead to greater utilisation but not to the extent that the substitution effect from lower relative prices of healthcare is greater than the income effect from lower income available to consume after paying for insurance. The income effect from paying for PHI premiums is non-negligible. In the sample used for this analysis, individuals with PHI pay, on average, a premium corresponding to 3.6% of their disposable income. Hence, premium payments are significant, suggesting that there should be an income effect.

The case of moral hazard appears different for PHI covering hospital care, which is free of charge when using the public system while it may require OOPE when using private care. When PHI for hospital care is not fully comprehensive and requires co-payments, private care will incur greater OOPE than public care. Yet, because all individuals can access care in public hospitals, PHI does not increase the risk of OOPE, as using the fee-attracting private hospital channel always includes an active choice. Hence, only this choice to use private care leads to greater exposure to OOPE. Buying PHI may entail some ex-ante moral hazard for PHI for private hospital care, as some individuals may purchase PHI because they plan to use private instead of public care. In addition, there may be some ex-post moral hazard if having PHI increases the probability of using the private instead of the public hospital.

Finally, some buyers of more but not fully comprehensive coverage may also be unaware of the actual OOPE they face when using private hospitals and may overestimate the financial protection that their PHI provides. Jeon et al. (2012) find that some older Australians with multiple chronic conditions purchase PHI due to an ill-founded understanding of the costs involved and because they are motivated by values of self-reliance and independence, as they wish not rely on government support for healthcare. This leads to them to use more private care, while being unaware of the limits of coverage that their PHI provides. Our results align with this type of relative over-reliance on PHI due to an unawareness of the amount of OOPE required to be paid by individuals with PHI when using private care.

### Implications for policy

Policy makers may be concerned about OOPE if it creates a significant burden for higher-need or poorer individuals. They may, therefore, consider that PHI could mitigate this burden, given that PHI as an insurance should, in principle, shift uncertain and variable OOPE to certain and fixed PHI premiums. We show that, in Australia, the share of income spent on OOPE by individuals without PHI is moderate, about 1% for lower need individuals and about 2% for higher need individuals who require hospitalisation. The OOPE share among PHI holders is higher at all levels of need and utilisation, but it is still only about 2% for those with lower need and about 4% for those with higher need and hospitalisation. Overall, the average levels of the share of income spent on OOPE that we observe in our data remain much below 10%, which is commonly used as an indicator of high burden of OOPE (Baird, 2016). Hence, while the OOPE share of individuals with PHI is consistently about double that of individuals without PHI, even for the group observing the highest OOPE share of income, the burden appears small because the OOPE share for individuals without PHI is very low and twice this share may still be considered small.

As attaining PHI is, notwithstanding tax incentives for purchasing PHI, an individual choice, this may not appear to be a great policy concern, even if PHI encourages (over-)use of healthcare services, for example, through an increased utilisation of PHI-subsidised, low-value treatments, or physician-induced demand (Chalmers et al., 2019; Duckett & Nemet, 2019b; Dahlen et al., 2014). Individuals with PHI can always access the public system in Australia, where OOPE appears to be less of an issue.^10^ Furthermore, PHI for ancillary services may help address unmet need for services that are not provided in the public system (Dennis et al., 2021). That some OOPE is required in these cases may be unavoidable and, in some cases, even desirable, particularly if the overall level of OOPE is moderate.

However, our results imply more of a policy concern when considering the burden of OOPE across the income distribution, as described in our Engel curves. That is, the OOPE share is much higher for lower-income individuals, both with and without PHI, and the increase in OOPE due to greater need and utilisation is steeper for poorer individuals. Furthermore, the difference in the OOPE share between individuals with and without PHI is greatest and increases more strongly with need and utilisation among poorer individuals. Hence, for poorer individuals, OOPE presents a significant burden, and PHI does not appear to mitigate this burden. We therefore argue that, from an equity perspective, OOPE is a policy concern in Australia, and our results suggest that PHI does not appear to help solve it.

### Limitations

Our analysis has limitations. First, we describe OOPE by PHI status but do not identify a causal effect of PHI status on OOPE. PHI holders may have a greater preference for healthcare than individuals without PHI. These preferences could play out stronger (i.e., inducing healthcare services use that requires OOPE) when they interact more frequently with healthcare providers because of their chronic conditions or when being hospitalised. Future work that identifies a causal effect of PHI on OOPE would be informative.

**Fig. 4:**
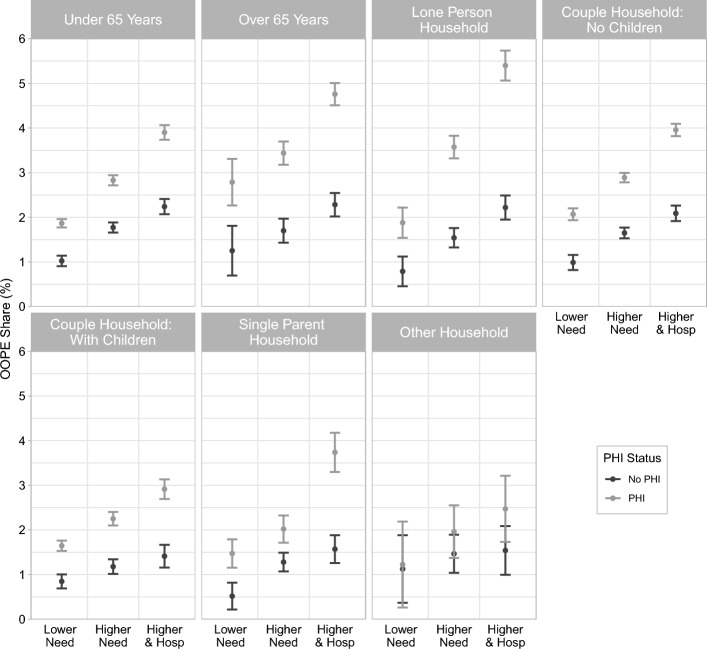
Heterogeneity by Age and Household Type. *Notes* Heterogeneity results are produced for sample subsets based on age and household type. Predictions of the conditional mean of the OOPE share are produced using the random effects model. Error bars around the point estimates show the $95\%$ confidence intervals. Individuals in the ‘Under 65 Years’ cohort are aged between 40 and 65 years. All household types contain less than five members

Second, we investigate OOPE and PHI at the individual level, but expenditure is measured only at the household level. The measure of the OOPE burden used here is the household OOPE divided by the household disposable income, while PHI status is an individual-level variable. To reduce the effect of this limitation, our sample did not include households with more than four individuals and includes control variables for household size and structure. In addition, Fig. 4 in the appendix shows that the described pattern of an increasing difference in the OOPE share between PHI and non-PHI individuals is observable for all types of household composition.

Third, a significant share of households has missing expenditure data in the self-completion questionnaire, with missing values ranging from 16 to 20 percent of the overall sample, depending on the wave and expenditure category. Because we estimate our results based on households that reported expenditure data, our results may be biased if non-response is not random. However, for our overall findings to be biased, the degree of non-response would have to differ between PHI and non-PHI holders and by their level of need and hospitalisation. That is, differences in non-response between individuals with and without PHI, which may be plausible because PHI holders are typically more educated, would have to increase with need and hospitalisation. While we do not find evidence that PHI and non-PHI individuals differ more strongly across our three categories based on these variables, our results may be limited by unobservable factors that determine non-response.

Fourth, our three categories do not capture all possible aspects of need and utilisation and are likely to miss unmet or undiagnosed need. While acknowledging that our measures of need and utilisation are imperfect, they allow us to define a gradient in need and utilisation and to study expenditure relative to this gradient.

Fifth, our study cannot determine whether buyers of PHI expect their PHI to lower their OOPE. Individuals buy insurance for various reasons and replacing variable expenditure (OOPE) with fixed insurance premiums may not be of great importance to insurance takers, even if this is the central purpose of insurance in economic theory. Determinants of the demand for PHI in Australia are probably only partly known. They will include factors such as avoiding long waiting times for elective surgery, peace of mind, the ability to maintain greater agency in health decisions, responses to fiscal incentives, and others.

**Fig. 5:**
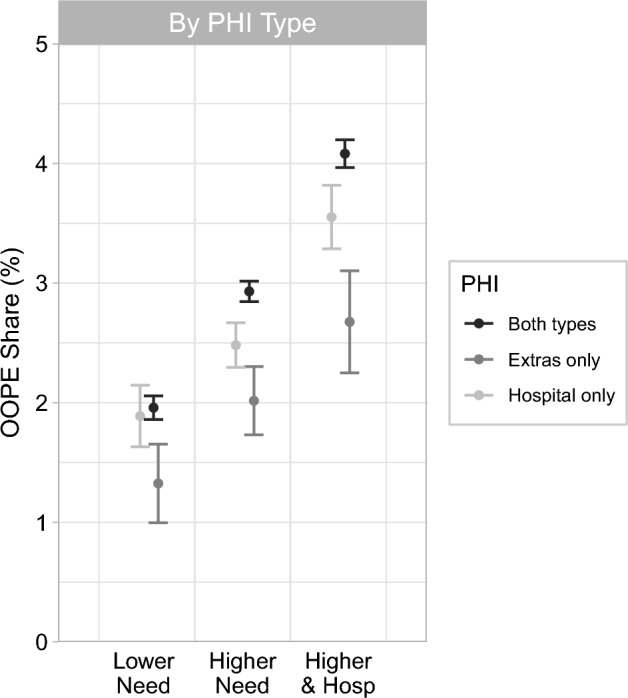
Random effects predictions by Need, Hospitalisation, and different types of PHI status. *Notes* Error bars around the point estimates display predicted 95% confidence intervals. Results correspond to the ‘Demographic & Health Controls’ condition in the main paper but include individuals with either only extras (ancillary) or only hospital cover, in addition to individuals with both types of cover

Sixth, we use a binary indicator of PHI for anyone with either both ancillary and hospital PHI, or neither. This means that we are combining PHI for two types of healthcare services, for which the role of PHI in reducing OOPE may differ. That is, ancillary insurance covers care not provided in the public system while care in private hospitals is also provided in the public system. Figure 5 in the appendix shows that the same pattern of increasing OOPE shares for individuals with PHI relative to those without PHI is observed for all possible combinations PHI (hospital, ancillary, or both). Hence, our results focussing on individuals with both types of PHI appear informative independent of the exact choice of our PHI variable. Furthermore, because we use a dichotomous variable, we do not know if our results are driven by individuals with lower coverage and higher deductibles or observed for all buyers of insurance. Because information on insurance coverage is not available in HILDA, this remains an open question for future research.

Seventh, we report the share of OOPE relative to an individual’s income, as typical in the literature on the burden of OOPE. However, PHI may still reduce the share of OOPE in overall expenditure on health, including OOPE and payments made by the insurers to providers. If such overall expenditure on health can be reduced due to PHI, this could benefit individuals who face lower total expenditure on health. Because our data do not include information on the amount spent by insurers, such patterns would need to be identified in future research.

## Conclusion

Many individuals in Australia purchase PHI. Some PHI holders and policy makers may consider that PHI is an insurance that protects against high OOPE, replacing uncertain OOPE by certain premiums, particularly when individuals have greater need or utilisation. Our results show that PHI is associated with a greater share of income spend on OOPE, and this share increases (in absolute terms) more strongly with need and utilisation for individuals with PHI than it does for individuals without PHI. Adverse selection and moral hazard may theoretically be argued to drive these results. Still, we do not find strong indications that they do, although they could be contributing to our findings.

We, therefore, argue that Australian PHI may not provide an insurance function in a classical sense of reducing exposure to variable and uncertain financial payments at the cost of the insurance premium. Although this observation is not necessarily central for PHI holders in their decision to buy PHI, it highlights that PHI in Australia may not provide this type of variance-reducing function. This, in turn appears informative for policy makers, as several policies are currently in place to increase PHI uptake. Even if these policies may primarily be motivated by other goals, such as improving access, they are not the only policy option to reach those goals, as, for example, access may also be increased within the public system.

We also find that the difference in the share of income spent on OOPE between PHI and non-PHI individuals, and the increase in this difference when need and utilisation increase, are greatest among poorer individuals. This finding highlights that OOPE reflects a significant burden for poorer individuals, particularly when they have PHI.

## Appendix

### Chronic conditions definition

The chronic conditions variable used in this research is a composite of two different measures from HILDA. The first measure is an individual reporting to be told by a doctor or nurse that they have any one of the following conditions: arthritis or osteoporosis, asthma, any type of cancer, chronic bronchitis or emphysema, type 1 diabetes, type 2 diabetes, depression, anxiety, other mental illness, heart disease, hypertension, or any other serious circulatory condition. The second measure is an individual reporting to have any long-term health condition, impairment or disability that restricts everyday activities, and has lasted, or is likely to last, for six months or more. Our definition indicates the individual has a chronic condition if they report in the affirmative to either of the two measures (Table 4 and Figs. 4, 5).

**Table 4:** Random effects coefficient estimates

Dependent Variable: OOPE Share (%)			
	Baseline case	Demographics	Demo & Health
Higher need	0.56^∗∗∗^	0.26^∗∗∗^	0.14^∗^
	(0.06)	(0.07)	(0.07)
Higher need and hospitalisation	0.94^∗∗∗^	0.51^∗∗∗^	0.32^∗∗∗^
	(0.09)	(0.09)	(0.09)
Lower need and PHI	0.44^∗∗∗^	1.11^∗∗∗^	1.04^∗∗∗^
	(0.07)	(0.07)	(0.07)
Higher need and PHI	0.71^∗∗∗^	1.40^∗∗∗^	1.36^∗∗∗^
	(0.07)	(0.07)	(0.07)
Higher need and hospitalisation and PHI	1.38^∗∗∗^	2.11^∗∗∗^	2.07^∗∗∗^
	(0.11)	(0.11)	(0.11)
Health status (First Lag)			− 0.01^∗∗∗^
			(0.002)
Age			0.02^∗∗∗^
			(0.003)
Log disposable income		− 1.91^∗∗∗^	− 1.87^∗∗∗^
		(0.07)	(0.07)
Health care card		− 0.44^∗∗∗^	− 0.72^∗∗∗^
		(0.07)	(0.07)
Year 12		0.27^∗∗^	0.34^∗∗∗^
		(0.09)	(0.09)
Certificate or diploma		0.19^∗∗^	0.27^∗∗∗^
		(0.06)	(0.06)
Bachelor degree		0.55^∗∗∗^	0.62^∗∗∗^
		(0.08)	(0.08)
Post graduate		0.72^∗∗∗^	0.79^∗∗∗^
		(0.09)	(0.09)
Couple with no children		− 0.16^∗∗^	− 0.15^∗∗^
		(0.05)	(0.05)
Couple with children		− 0.75^∗∗∗^	− 0.63^∗∗∗^
		(0.05)	(0.05)
Single parent		− 0.96^∗∗∗^	− 0.84^∗∗∗^
		(0.07)	(0.07)
Other household		− 0.95^∗∗∗^	− 0.87^∗∗∗^
		(0.11)	(0.11)
Time fixed effects	No	Yes	Yes
Remoteness region controls	No	Yes	Yes
Individuals	9754	9754	9754
Degrees-of-freedom	19260	19241	19239
Child vars jointly zero (*p*-value)	–	0.08	0.00
	**p* < 0.05; ***p* < 0.01; ****p* < 0.001		

The ‘Baseline Case’ model is without control variables. The ‘Demographics’ model includes control variables related to individuals and household demographics. The ‘Demo & Health’ model includes health status variables (age and the first lag of the PCS) in addition to the demographic controls. Parameters for the remoteness region of residence, and categorical variables indicating the number of children, and if any children are younger than five years, are not shown; however, the p-value for a null hypothesis that all child related variables are jointly zero is given in the bottom row. Coefficients are interpreted in comparison to the baseline case, given as: *Low Need*, no PHI, a lone person household, the highest level of education being less than year 12, and living in a major city. Standard errors are clustered at the individual level and presented in brackets below the coefficient estimates

## Footnotes

1 PHI can also provide coverage for private treatment in public hospitals, which may also require co-payments.

2 Note that data for the health module is collected in a self-completion questionnaire that has a higher fraction of non-response, an issue we discuss in the limitations section.

3 We would like to emphasise that while our PHI definition facilitates the interpretation of our results, our results do not depend on it.

4 The random effects model takes advantage of the longitudinal nature of the data by accounting for unobserved heterogeneity between individuals, providing estimates that are more efficient than those produced by a pooled ordinary least squares model, which does not account for individual level serial correlation within the error term. We cannot use a fixed effects model for our approach because the fixed effects model does not allow for the identification of the conditional mean of the dependent variable (the OOPE share) and can therefore only identify directional effects based on individuals who change their OOPE and their PHI status, need, or utilisation between different survey waves.

5 The term ‘statistical Engel curve’ indicates that theoretical economic assumptions are not built into the model.

6 As four is the maximum household size in the sample, three is the maximum possible number of children in a household.

7 We control for the effect of possible confounders assuming a linear effect of controls.

8 Hence, an individual-level random effect is not modelled into the error term.

9 Such greater use of ancillary healthcare services may also be beneficial for health, relative to individuals without PHI who forego allied healthcare even if they need it, with potentially negative long-term consequences (Dennis et al., 2021).

10 OOPE shares are lower and increase less with higher levels of need and utilisation among individuals without PHI who rely on the public system.
